# A Scalable Context-Aware Objective Function (SCAOF) of Routing Protocol for Agricultural Low-Power and Lossy Networks (RPAL)

**DOI:** 10.3390/s150819507

**Published:** 2015-08-10

**Authors:** Yibo Chen, Jean-Pierre Chanet, Kun-Mean Hou, Hongling Shi, Gil de Sousa

**Affiliations:** 1 IRSTEA – Centre de Clermont-FD, TSCF, 63173 Aubière, France; E-Mails: chenyibo.clover@gmail.com (Y.C.); gil.de-sousa@irstea.fr (G.S.); 2 Laboratoire LIMOS UMR 6158 CNRS, 63173 Aubière, France; E-Mails: kun-mean.hou@isima.fr (K.-M.H.); shi@isima.fr (H.S.)

**Keywords:** WSN, RPL routing protocol, 6LoWPAN, protocol evaluation, Internet of Things

## Abstract

In recent years, IoT (Internet of Things) technologies have seen great advances, particularly, the IPv6 Routing Protocol for Low-power and Lossy Networks (RPL), which provides a powerful and flexible routing framework that can be applied in a variety of application scenarios. In this context, as an important role of IoT, Wireless Sensor Networks (WSNs) can utilize RPL to design efficient routing protocols for a specific application to increase the ubiquity of networks with resource-constrained WSN nodes that are low-cost and easy to deploy. In this article, our work starts with the description of Agricultural Low-power and Lossy Networks (A-LLNs) complying with the LLN framework, and to clarify the requirements of this application-oriented routing solution. After a brief review of existing optimization techniques for RPL, our contribution is dedicated to a Scalable Context-Aware Objective Function (SCAOF) that can adapt RPL to the environmental monitoring of A-LLNs, through combining energy-aware, reliability-aware, robustness-aware and resource-aware contexts according to the composite routing metrics approach. The correct behavior of this enhanced RPL version (RPAL) was verified by performance evaluations on both simulation and field tests. The obtained experimental results confirm that SCAOF can deliver the desired advantages on network lifetime extension, and high reliability and efficiency in different simulation scenarios and hardware testbeds.

## 1. Introduction

Thanks to the rapid advances in the WSN domain (e.g., miniaturization of WSN nodes), Precision Agriculture (PA, also called precision farming in certain contexts) has started to emerge as a new trend in the agricultural sector in the past few years. Generally speaking, a PA system concentrates on providing ways for observing, assessing and controlling agricultural production processes, and covers a wide range of uses from herd management to field crop production [1]. For example, the site-specific crop monitoring application involves numerous different issues. One of them is environmental data collection, such as monitoring soil, crop and climate in one/multiple fields, which are separated by parcels. Furthermore, a Decision Support System (DSS) can use these sensory data for promising treatments analysis of a whole field or a specific parcel. Meanwhile, different actions can be carried out under the guidance of the analysis results and subsequent monitoring, for instance, adjusting in real-time operations such as fertilizer, lime and pesticide utilization, tillage, or sowing rate [2].

PA and WSN applications combine an exciting new topic of research that will greatly improve quality in agricultural production, water management and at the same time will significantly reduce the cost and environmental impact of agricultural production. We note that a WSN is able to provide precise cultivated field information in real-time, and help reduce resource usage to minimize environmental pollution due to overuse of fertilizers, pesticides, *etc*. Furthermore, the open standardized protocols, the ease of deployment and system maintenance open a door to introduce the next generation of WSN—Internet of Things (IoT) technologies in PA. In addition, the cultivated field sensory data can be also used in supply chain management [3].

Most of the current agro-environmental monitoring applications transmit the real-time data in wireless networks through a M2M (machine to machine) support platform. Some systems utilize SMS, Web, WAP patterns, so that the terminal can receive the information to monitor the production. However, these earlier researches count on composite Web applications on the server side. Thanks to the 6LoWPAN adaptation protocol [4], smart objects are capable of efficiently connecting to the IPv6-based internet [5]. With the other protocols, such as RPL and Constrained Application Protocol (CoAP) [6], it is hoped they will improve the autonomous capability of the resource-constrained WSN nodes in PA systems, and enhance the performance of the whole network.

Therefore, as the development of IETF standardization in the fields of IoT and Web of Things (WoT), their use cases will be not limited in the current applicability coverage (e.g., home automation [7,8], commercial building automation, industrial automation, urban environments [9]), and PA application will become a significant and promising one in the near future. Meanwhile, adoption of IoT technologies in PA applications has to tackle the challenges due to the various constraints of specific WSN nodes, their deployment environment and applications. Thus, the WSN of a PA system is a Low-power and Lossy Network (LLN), and RPL [10] has been considered as a natural solution for routing issues in Agricultural LLNs (A-LLN) in our previous work [11].

According to the ROLL Working Group (WG) charter, RPL is designed as a general widespread use routing protocol, independent from the rest of the protocol stack through proposed self-organization mechanisms–topology structures. However, from the evaluations of [12] and the applicability analysis of [11], the original RPL protocol and its existing implementations cannot meet the Quality of Service (QoS) requirements of real world A-LLNs deployments, such as resource constraints, transmission range, network lifetime, automatic topology adjustment and resistance of highly dynamic environment caused by obstacles, adverse weather or vegetation growth [13]. To overcome the above challenges, an optimization mechanism for RPL, introducing context-aware features (*i.e.*, remained energy, hardware/software reliability, link quality, *etc.*) into the Objective Function (OF), has been taken into account in our proposed Routing Protocol for A-LLNs (RPAL) model. The RPAL routing algorithm is devoted to the application and network infrastructure of PA, experimented and evaluated as a part of IoT protocol stack with 6LoWPAN and CoAP in both simulation and field tests.

In what follows, the routing challenges and design issues in A-LLNs are described in Section 2. In Section 3, the RPL protocol is briefly presented, and then in Section 4 the SCAOF is presented, evaluated and the results are discussed in the simulation scenario (Section 5) and hardware testbeds (Section 6). Finally, the conclusions of this paper are mentioned in Section 7.

## 2. Integrating Precision Agriculture and IPv6 Low-Power and Lossy Networks

Precision agriculture is considered as a use-case scenario based on the LLN framework. A-LLN, which is the integration of PA and LLN, presents the intrinsic characteristics of WSNs used by typical agro-environmental monitoring systems. On the one hand, LLNs exclusively use IPv6 which is the key component of IoT [14]. On the other hand, WSN will be the most popular and practical solution for ICT in agriculture [2] when wired network deployment cannot cover the cultivated fields, grazing lands and monitored sites, which may reach several tens of hectares, with controllable costs. Thus, an efficient A-LLN routing solution can push the WSNs-based application of Information and Communications Technology (ICT) in agriculture to IoT. As a proprietary type of LLN, the RPL applicability analysis of A-LLN inspired by [15] has been elaborated in [11].

As a new trend in the agricultural sector in the past few years, ICT in PA concentrates on providing various techniques to cover a wide range of applications, from herd management to field crop production [16]. In most of the cases, PA is always referred in the issues of site-specific crop management and includes three aspects:

− Environmental monitoring (e.g., temperature, light intensity, atmospheric pressure, soil moisture or air humidity, UV intensity, strength and direction of wind, rainfall, gases, pH of dust or rainwater, and heavy metals) in a field which is separated by some complete parcels;
− Utilizing DSS to obtain possible treatments analysis, which can be applied for field-wide or specific parcel;
− The methods of adjusting corresponding operations in real-time, such as fertilizer, lime and pesticide utilization, tillage, irrigation, and sowing rate.

In the current PA domain, diverse applications have been developed and wireless sensor motes are scattered throughout the cultivated fields to monitor needed data, such as soil moisture, atmospheric temperature, light and wind strength, hours of sunshine, rainfall measurement, and humidity of the leaves. These sensory data are the basic component of a DSS that enables resource (e.g., water, herbicide, pesticide, fertilizer, *etc.*) optimization [17], disease detection and development prediction, pest control, frost protection [1], avoiding environmental pollution, intruder detection, yield prediction, fire detection, *etc.*

The A-LLNs infrastructure of a PA system is normally composed of 20~50 devices which are typically interconnected using wireless technologies (e.g., ZigBee PRO or Wi-Fi protocols [2,18,19,20]) with a backhaul network providing connectivity to “command-and-control” management software systems (e.g., DSS) at the data processing center of an experimental farm. Note that the current researches of PA system [18] are mainly dedicated to the needs of modern agricultural paradigm shift, remote monitoring/control, asset tracking and distance diagnosis.

With the help of wireless connectivity and access from A-LLN nodes, users can collect significantly increased amounts of information and remotely manage a larger number of control points. One A-LLN is the smallest part in this scenario and it would contain and be equipped with three kinds of key nodes that are outlined below. Notice that the appellation of A-LLN sensor node is a substitution of WSN nodes. Its main purpose is also to offer measurement sensory information.

Considering the deployment of A-LLN nodes, they will be manually deployed in a monitored field within defined topological constraints under the assistance of GPS. In addition, a number of trades-offs have to be considered: equipment maintenance costs, network density, network lifetime and existing IP network infrastructure [21].

It is usually the case that A-LLN traffic patterns are highly asymmetric, where the majority of the traffic volume generated by the A-LLN sensor nodes typically goes through the LBRs, and is directed to the data center servers, in a Multipoint-to-Point (MP2P) fashion. Meanwhile, the data center server can generate Point-to-Multipoint (P2MP) communication through LBRs to configure A-LLN devices or initiate queries, and use unicast and multicast to efficiently communicate with a single A-LLN sensor node or LC node, or groups of these devices. Additionally, based on the LLN concept defined by ROLL WG, a layered architecture protocol stack installed in the A-LLN devices is friendly for any data link layer, such as the 802.15.4 and 802.11 families.

In other words, periodic sensor monitoring dominates the traffic generated by A-LLN nodes, and PA applications normally do not have hard real-time constraints as the exchange of sensory data is slow and smooth in most of the time. Furthermore, A-LLN sensor nodes are always configured to run in Sleep&Wakeup mode, and they are often subject to bounded latency and stringent reliability service level requirements. From the perspective of routing requirements, A-LLN requires both of efficient MP2P and P2MP communications. Besides, the routing protocol operating in A-LLN deployment needs to provide good scalability with network size and number of forwarding hops. More supplementary mechanisms, such as timely loop detection and resolution, broken link repair and QoS-aware routing path selection and optimization are still important due to the highly dynamic environment for A-LLN. Last but not least, A-LLN scenario can be regarded as a complement to the application-specific routing requirements defined and analyzed by IETF ROLL WG, such as urban, industrial, home automation, and building automation LLNs. Advanced metering infrastructure (AMI) (or smart grid) documented and stated more exhaustively in [22,23] has greatly inspired our work. Therefore, the work of this article is also a further extension of our previous work [20].

## 3. IPv6 Routing Protocol for Low-Power and Lossy Networks: State-of-the-Art

RPL is a standard designed exclusively for routing in LLNs with the expectation of joining thousands of conventional WSN nodes in the network. It supports three traffic patterns: MP2P, P2MP and point-to-point (P2P). The basic idea of RPL is that the high degree of autonomy in the WSN nodes level through building a Destination Oriented DAGs (DODAGs) rooted towards one sink.

In the RPL-based network, three types of control messages are used. DODAG Information Object (DIO) messages (sent in multicast way) are used to construct and maintain upwards routes of the DODAG for MP2P traffic pattern. Moreover, downward routes (for P2MP) are managed by Destination Advertisement Object (DAO) messages that are sent by router nodes and used for the propagation of routing tables. Another common message is DODAG Information Solicitation (DIS) that can be sent by any node in the RPL to solicit DIO messages from its neighborhood to update routing information. In the RPL specification [10], there are more detailed explanations that the reader can find and comprehend, and a better organized and clarified statement of RPL in [24,25].

ROLL WG has a long vision for the development of RPL routing protocol. OFs introduce high flexibility into its framework. DODAGs can be optimized according to a specialized OF and be identified by an Objective Code Point (OCP), which indicates the dynamic constraints and the metrics (listed in Table 1) [26]. Therefore, RPL is easily to be adapted to meet the requirements of different LLNs and application scenarios thanks to the flexibility and scalability of OFs. Starting from the documented OF0 in [27], numerous contributions have been undertaken to achieve QoS aware routing in various LLNs use cases [7,23,28], and to test RPL framework by modeling and experiments.

**Table 1:** A list of routing metric/constraint objects for RPL.

Routing Metric/Constraint Objects	Description
**Node State and Attribute**	CPU, Memory, congestion situation
**Node ENERGY**	Power mode, estimated remaining lifetime
**Hop Count**	Number of hops
**Link Throughput**	Maximum or minimum value
**Link Latency**	Sum of all latencies, pruning links higher than certain threshold
**Link Reliability**	Packet reception ratio, BER, mean time between failures... Link Quality Level (LQL); ETX
**Link Color**	10-bit encoded color to links, avoid or attract specific links/ traffic types

RPL is designed to be widely applicable, and it provides a set of available configuration options. The RPL specification [10] presents a number of design choices and configuration parameters that are references to build a more efficient RPL implementation. In [29], an overview and evaluation profile (*i.e*., two use cases: a small outdoor nodes deployment for building automation and a large-scale smart meter network) of RPL are presented. Furthermore, the above RPL implementations usually require a protocol stack as a structure to build a low-power and reliable mesh network that can be fully integrated into the Internet. To the best of our knowledge, ZigBee IP [30], 6TiSCH [31], OpenWSN [32] and Contiki uIPv6 [33] are the current four most promising and viable protocol stack solutions for IoT including RPL as default routing protocol. In addition, the Contiki COOJA [34] platform is adopted by the authors of [35] to evaluate network overhead, throughput and end-to-end delay for different network sizes. For real world devices, ContikiRPL is the first RPL implementation. It has been built into Contiki OS as a default routing protocol. Moreover, Contiki is a comprehensive platform including simulation, experimentation, and evaluation of IoT protocol stack.

The flexibility of RPL can tackle the specific requirements from the characteristics of A-LLNs. On the one hand, ROLL WG has documented a number of RFCs to specify the OFs [36], supported scenarios [5], design guidelines and requirement definitions of the final protocol [7,23,28], routing metrics [37], energy optimizations and stability mechanisms [38,39], and preliminary protocol test results [40]. On the other hand, more attention has been paid to the specific mechanisms of RPL and more practical issues in real world environment are considered.

To start with the key components of routing in LLNs, namely, routing metrics and OF in RPL defines how to translate one or more metrics and constraints into a rank [41] which is also similar to gradient-based routing [42]. Currently, the standard RPL framework has two OF algorithms: OF0 [27] and Minimum Rank with Hysteresis Objective Function (MrhOF) [41]. The latter is more practical and is preferred by the existing RPL models. The authors in [43] propose a passive probing and cache management solution to solve the drawbacks (e.g., hidden available parents) induced by using Expected Transmission Count (ETX) as link cost estimation, but their proposal aggravates the hotspot problem so the cumulative phenomenon could influence the final network lifetime, that is, if energy-constrained LLN nodes that are close to the data sink have a tendency to die earlier, parts of the network will be left completely unmonitored and become network islands. Thus, the authors of [38,44,45] consider joining multiple contexts to conduct routing in LLNs. In [38], the authors present an energy-aware and resource oriented enhanced version of RPL, called Resource Oriented and Energy Efficient (ROEE) RPL. In [46], Liu *et al.*, present a new RPL OF and routing algorithm (*i.e.*, LB-RPL) to construct a load balanced DODAG. Notice that they adopt a distributed and non-intrusive fashion to detect the workload imbalance. Another reference which inspires our work [47] offers a solution to increase the network lifetime of biomedical WSNs based on a new Energy-Aware OF (EAOF) with computation of both ETX and remaining energy metrics for RPL routing on each LLN node. More examples of the utilization of routing constraints are stated in the RFC document [26].

Additionally, in [37], the authors reveal the drawback of using a single metric, and the possibility and requirements of adopting composite metrics. Generic rules for metrics composition in lexical and additive manners have been defined to achieve convergence, optimality and loop-freeness for RPL. The authors also offer a conclusion: the lexical approach is less restrictive, and the additive manner is more demanding in the mathematical formulation but has more flexibility which can satisfy various QoS requirements according to user demand. Moreover, the authors in [37,48] elaborate how to apply composite RPL routing metrics to satisfy different application needs and LLNs QoS requirements. They also specify the ways to combine primary RPL routing metrics (Table 1) and prove their proposal by the theoretical framework of routing algebra formalism, and evaluate the performance of their approach quantitatively through the verification of the simulation scenarios.

In real world A-LLNs, using a cross-layer philosophy [49] is a solution to increase the efficiency of the designed protocol stack. The authors of [50] mutually pool the entire protocol layers of a uIPv6 stack. This method is referred as “vertical calibration” in [51] because their proposal strives to find the optimal settings from a global view of the protocol stack, namely from the application level to the MAC level. Moreover, the authors of [52] adopt conventional cross-layer optimization, and propose stateless multicast RPL forwarding (SMRF) to improve “Multicast Forwarding Using Trickle”. Meanwhile, as tight relations with adjacent layer, the researchers of [53,54] present their contributions for RPL optimization dedicating to the interactions between routing layer and IEEE802.15.4 MAC standard. Furthermore, the utilization of cross-layer approach is not limited in the above scope. The authors of [27,30] indicate that a cross-layer protocol combining MAC layer and hardware solutions can achieve high energy efficiency.

Last but not least, most of the existing evaluation works for RPL-based proposals adopt simulation scenarios, and the works of testing RPL on testbeds (See Table 2) are still scarce. As a promising protocol, RPL and its enhanced model require more experiments using real world WSN testbeds, especially in outdoor environment. Notice that the real world problems are the essential concerns for RPL, and motivates our following contribution.

**Table 2:** Existing real world testbeds for the evaluation of RPL protocol.

Reference	Platform Name	Size of Network	Indoor/Outdoor	Hardware Platform	Evaluated RPL Model
[55,56,57,58]	Indriya testbed	135 WSN nodes	Indoor	TelosB nodes with Arduino	ContikiRPL-->ORPL
[59]	SensLAB platform of INRIA Lille	100 WSN nodes	Indoor	WSN430 boards with TI CC2420 radio chip	ContikiRPL
[60]	TinyRPL testbed	51 WSN nodes	Indoor	TelosB motes	TinyRPL and BLIP
[61]	PLC testbed on INRIA	6 PLC nodes	Indoor	CC2420	RPL for PLC network
[24]	Multi-hop topology testbed	30 WSN nodes	Indoor	TelosB motes	ContikiRPL

## 4. Enhanced Objective Function for Routing in Agricultural Low-Power and Lossy Network

The main idea of our proposal is a routing solution of A-LLNs scenario based on RPL’s OF with a combination of path weight value calculating algorithms and composite routing metrics, considering of elementary metrics defined in [26].

In real world systems, the dynamic environment causes various issues, for example, the complexity of after analysis (*i.e.*, unavoidable radio interference, noise, *etc.*) and unpredictability of system problems (*i.e.*, hardware, OS, *etc.*), which are the main challenges for protocol design and evaluation. In addition, A-LLN devices have resource constraints, such as low memory capacity, low computation capability and low energy budgets. Unfortunately, the literature on improving and adapting RPL for specific real world applications is very scarce, if we consider that only the smart grid, industrial automation and E-health areas have been addressed, not to mention agricultural WSNs.

To the best of our knowledge, our study is the first one to extend the conventional WSN of ICT in agriculture to the concept of LLNs [11], exclusively supporting IPv6 that is main ingredient of an IoT platform. Moreover, the main purpose of this contribution is to enhance the applicability, robustness, and scalability of RPL for A-LLNs. Thereby to adapt RPL to the precision agriculture application, we start with a simple OF by considering energy-aware metrics. Then, we propose a scalable OF with composite RPL routing metrics.

For dealing with the intricacies presented by A-LLN, one routing metric is not sufficient to represent the QoS requirements defined by a PA application. Meanwhile, since the PA application needs its underlying A-LLNs respecting to a required level of QoS (delay, reliability, energy consumption and packet loss) a set of rational routing metrics are elected. We use a RPL routing metric composition approach [37] to combine different primary routing metrics to satisfy the needs of A-LLNs’ characteristics and limitations. The corresponding mechanisms are designed as accessories of RPAL model to capture the concerned effects (e.g., link reliability, remaining energy, *etc.*).

### 4.1. Energy-Aware Metrics and Objective Function of IPv6 Routing Protocol for A-LLNs

The energy-aware metrics and OF of IPv6 Routing Protocol for A-LLNs (RPAL) are designed with the following purposes: to mitigate the effects of packet losses and electromagnetic interferences in A-LLNs when they are deployed in harsh environments (e.g., field tests); to balance the energy consumption and prolong the A-LLNs lifetime. As an enhanced version of RPL framework, RPAL can identify and mitigate the energy hole problem. In other words, this proposal is able to avoid congestion or route overuse that will lead to the premature death of A-LLN nodes. Two metrics, namely ETX and RE, which can be organized in a lexicographic manner [37], can ensure the preferred parent selection is more efficient.

On the one hand, integrating ETX metric can guarantee that RPAL routers can select the node within enough reliable paths to DODAG root to be its preferred parent. On the other hand, Remained Energy (RE) metric on each node with energetic considerations in the construction of DODAG and preferred parent selection is also combined. Under the help of RE estimated method (*i.e.*, PowerTrace model [62]), RPAL model can promote energy balance while still choosing reliable and efficient routing paths. Moreover, RPAL OF algorithm makes use of ETX and RE metrics efficiently to increase A-LLNs lifetime.

**Figure 1:**
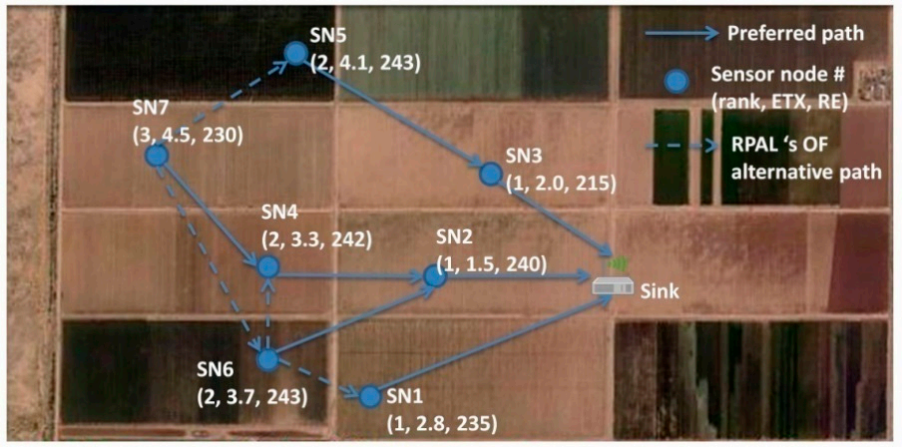
A-LLN scheme. SN2 has to forward the messages from SN4, SN6, and SN7 to the sink. Consequently, it will become inactive due to the hotspot issue.

One A-LLN with seven sensor nodes and one sink is located as shown in Figure 1 where the A-LLN nodes are equipped with agricultural sensors, periodically send monitoring data to the sink. We firstly assume that this is a stable network and the links are symmetric. Only SN1, SN2 and SN3 are in the radio range of the sink and can forward the messages from the other four nodes. Each node has three attributes: Rank, link ETX, and RE. If MrhOF and ETX metrics are used to build routing paths to the sink, SN4, SN6 and SN7 will choose SN2 as their best parent due to its lowest ETX. Consequently, SN2 will deplete its energy faster and become inactive, and the network cannot respond properly to subsequent messages. To overcome this hotspot issue, the RPAL OF algorithm takes the available energy into account. Namely, two routing metrics, ETX and RE of each node are used to compute the best path to the sink. Here is the principle of preferred parent selection: each node chooses its forwarders from its neighbors which have feasible links to the sink. Then from this subset, two nodes having maximum RE are selected to become the best and backup parents of this node. Maximum path residual energy calculation like ELR and MMBCR [63] is used in RPAL OF. Essentially, this metric can avoid the routes traversing the nodes which have the least battery capacity among all possible routes [64].

A DODAG Information Object (DIO) control message [10] is the carrier of ETX and RE metrics. The ETX value is measured by the link layer. ETX of a sink node (SN1 in Figure 1) is zero and the energy of the sink node is constant at 100%. The RE value is represented in percentage values rather than in joules units, so only one byte is required. ETX and RE are reflected in the metric containers [10] defined by ROLL WG and embedded in the options part of the DIO format.

**Figure 2:**
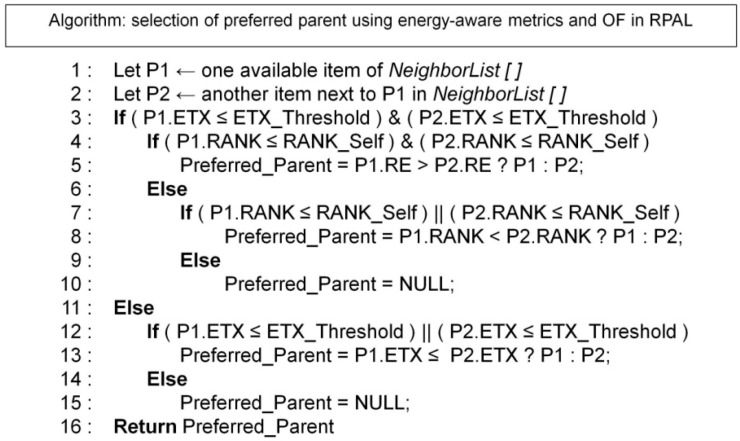
RPAL OF selects the neighbor with the viable ETX and the highest RE to be the preferred parent.

Figure 2 presents the RPAL OF algorithm that is also an extension based on the ContikiRPL OF [65]. It assumes there are at least two neighbors/parent candidates for this router/leaf node. The parameters ETX_Threshold and RE_Threshold are re-configurable and dependent on A-LLN’s application. The ETX_Threshold represents the maximum level of ETX value considered as a best parent candidate. It is pertinent to packet E2ED, and depends on the application QoS requirements. The RE_Threshold is the minimum difference of RE for a node to trigger the best parent switch. These two values introduce a configurable level of hysteresis in order to conduct the preferred parents updating, so RPAL OF could be configurable for the specific PA application. The algorithm can repeatedly search for the node with the highest least energy node routing path among all the routes with minimum ETX. Finally, to avoid loops, each node rules out the neighbors with greater rank from being its optional parents.

### 4.2. Scalable Context-Aware Objective Function with Composite Routing Metrics

By adapting RPL to PA applications, basically, A-LLNs are able to support environmental monitoring and remote control applications in the real world environment. Further efforts are needed for an investigation of the routing metric combination and proving its efficiency (*i.e.*, loop-free, optimality and convergence). For this purpose, Scalable Context-Aware Objective Function (SCAOF) is a lightweight solution to properly utilize a set of metrics in the RPAL model.

Apparently, using a composite of the primary routing metrics ETX and RE is not sufficient to mitigate the intricacies of real world A-LLNs. For example, an environmental monitoring application in the experimental field is loss and delay tolerant. Thus, a tradeoff might be performed in these two metrics. Namely, an ETX routing metric can have a higher threshold taking the retransmission mechanism into account. If A-LLN sensor nodes are required to support responding for the sensing data queries or remote control actuators in a parcel of field, the communication reliability is more important than any other performance perspective, including energy consumption. SCAOF in RPAL is a specific algorithm based on a routing algebra and routing metrics composition approach for real world A-LLNs. Moreover, additional resource-aware metrics are combined into the RPAL model through SCAOF, which can provide a more practical upward routing path after integrating context-aware resources into the best parent decisions.

In [66], its authors provide a more detailed theoretical background (*i.e.*, principles and basic theorems of routing algebra), and methods to prove and evaluate the designed composite metrics. However, an abstract RPL model in JSIM network simulation [37] could only present the performance of combining primary metrics at a restricted level. Therefore, SCAOF will be validated in cross-level simulation using Cooja and evaluated in a hardware testbed, which can introduce more precise properties of realistic wireless network scenarios. Furthermore, our contribution is able to generalize the composite metric manner for application in real world systems.

The composite routing metric solution follows the routing algebra stated in [67]. One routing metric defined by ROLL WG [26] can be formally represented as a quadruplet (S,⊕,ω,≾) path weight structure, where Ѕ is the set of paths, ω is a OF that maps a path or a link to a weight value, ≾ is a special order relation and ⊕ is the path concatenation operation. For example, where ω(α)≾ω(β) means “path α is lighter (better) than or equal to path β”, ≾ provides a total order of weights and it can cooperate with ω to capture different path and node characteristics (*i.e.*, delay, bandwidth, hop count, link reliability, energy consumption, *etc.*). The essential purpose of routing algebra is to use appropriate metrics for routing packets along the lightest/optimal path according to the decisions based on an ordered path set. Furthermore, it also introduces two primitive properties of routing metrics composition: monotonicity and isotonicity.

Briefly speaking, if a selected metric is monotonic, then the network topology made by this metric can be free of loops. In other words, the monotonic property could ensure network convergence for a routing algorithm. The isotonicity property essentially affects the order of the path weights and could guarantee the convergence is optimal for distance vector protocols like RPL. According to the work of Yang *et al.* in [67,68], the primary routing metrics have been investigated and proved their monotonicity and strict isotonicity, which can insure the optimality, consistency and loop-freeness for any routing protocol type. Nevertheless, some other routing metrics have to be test carefully.

Gouda *et al.* take the lead in defining the lexicographic and additive routing metric compositions in [69]. The lexical metric composition of two routing metrics $\left(\text{S},⊕,{\text{ω}}_{1},{≾}_{1}\right)$ and $\left(\text{S},⊕,{\text{ω}}_{2},{≾}_{2}\right)$ can be considered as the lexicographic composition relation ${≾}_{\text{lex}}$ over the ordered pair ${≾}_{1},{≾}_{2}$. If and only if, for every link pair 〈α,β〉 in S, they can satisfy the mapping of weight pairs $\left({\text{ω}}_{1}\left(\text{α}\right),{\text{ω}}_{2}\left(\text{α}\right)\right)$ and $\left({\text{ω}}_{1}\left(\text{β}\right),{\text{ω}}_{2}\left(\text{β}\right)\right)$ in the Cartesian product of the weight value sets for these two routing metrics ${\text{W}}_{1}\times {\text{W}}_{2}$. The additive composition relation ${≾}_{\text{add}}$ over the set ${\text{W}}_{1}\times {\text{W}}_{2}$ can be simply defined as: $\left({\text{ω}}_{1}\left(\text{α}\right),{\text{ω}}_{2}\left(\text{α}\right)\right){≾}_{\text{add}}\left({\text{ω}}_{1}\left(\text{β}\right),{\text{ω}}_{2}\left(\text{β}\right)\right)$ $\Leftrightarrow \;{\text{ω}}_{1}\left(\text{α}\right)+{\text{ω}}_{2}\left(\text{α}\right)\leq {\text{ω}}_{1}\left(\text{β}\right)+{\text{ω}}_{2}\left(\text{β}\right)$.

#### 4.2.1. The Problem Statement of Energy-Aware Routing Metric Composition

SCAOF can furnish the information and network characteristics with the routing metrics but it needs a new DIO carrier format. Meanwhile, the selected composite metrics of SCAOF should be suitable for the A-LLNs’ requirements as well as the monotonicity and strict isotonicity so the RPAL protocol can converge to optimal paths in a loop-free topology.

In [41,68], ETX has been proved to be strictly isotonic and monotonic. As we have already considered energy-aware routing metrics with ETX and RE, in fact, if ETX is calculated as the first metric, then it will dominate the path selection regardless of the path weights of the remaining metrics. Namely, the lexical metrics composition will take the second metric RE into account only if more than one path mapping has equal/less than the weights of ETX. In this case, essentially, the energy cost of retransmission is considered firstly, but if only a few paths are under a defined low threshold, they will have more traffic load than the others. Thus, a combination of ETX and RE in a lexicographic manner cannot completely solve the hot spot problem but can mitigate the consequential network disconnection issue.

The basic purpose of energy-aware routing is to increase of the network lifetime [70]. The RE indicating the lowest energy level in the path is concave and max-min criterion. If we define ω(α) to reflect the RE of a link α, and ℋ is the node set that constructs link α and i is an end node of this link, we can assume that $\text{ω}\left(\text{α}\right)=\text{min}\left\{{\text{RE}}^{\text{i}}|\text{ i}\in ℋ\right\}$ and ${\text{RE}}^{\text{i}}=\frac{{\text{C}}_{\text{now}}^{\text{i}}}{{\text{C}}_{\text{max}}^{\text{i}}}\leq 1$. If one node tries to select a forwarding path between α and β, the order relation ≾ is max{ω(α),ω(β)}, namely the “more than or equal” relation over real numbers. Thus, assuming ω(α)<ω(β), it may happen that ω(α)<ω(β)≤ω(γ) in which a special case: ω(α⊕γ)=max{ω(α),ω(γ)}=ω(γ)= max{ω(β),ω(γ)}=ω(β⊕γ). Thus, this RE routing metric is not strictly isotonic.

Moreover, although the RE metric of path can avoid the most fragile routing path, it is not good enough when sensor connectivity and coverage are considered [37]. Especially, when the RPAL model cannot capture sufficient and opportune energy-related attributes of all the routing paths, the parent selection may not be optimal due to the loss and delay of DIO control messages. Furthermore, as the aggregation rule of this RE metric is concave (min.), it only can be used in the lexicographic combination manner and additionally it limits the scalability of the RPAL objective function.

#### 4.2.2. Designing Combinable Energy-Aware and Resource-Aware Routing Metrics

Based on the above discussions, the RE metric has to be adapted to an additive routing metric then it should be strictly monotonic and isotonic to satisfy all the assumptions of theorems proposed in [37]. The selected additive RE metric is based on summated metric values of the links in the corresponding routing path. For example, we assume one path α and its length is n hops, then the weight of path α indicates the energy on average for all traversing nodes:

(1) $$\omega \left(\alpha \right)=\frac{\sum_{1}^{n}\frac{1}{Rem.Energy^{i}}}{n}$$

Note that the previous RE needs to be transformed to (1/RE), and (1/RE) will have the same metric range in the required granularization, order relation, metric operator as ETX. Like the guidelines shown by [37], Table 3 depicts two adopted metrics and one derived form of RE. Moreover, this routing metric cannot work alone since it also needs the hop count information, and three rules have to be clarified: (1) If two paths α and β have the same length n and all the traversing routers are battery-powered without the effects of energy harvesting devices, the path with higher average energy will be preferred; (2) If α and β have the same average energy value, then the path with the lowest length (or number of involved nodes) will be preferred; (3) If α and β have different average energy values and path lengths, then the routing decision should be made with the cooperation of ETX metrics.

**Table 3:** Descriptions of the ETX, RE and derived RE routing metrics.

Adopted Metrics	Domain	Aggregation Rule	Order Relation
ETX	[1, 512] × 128	Additive	(<) ➔ ([1, 512], “+”, “<”)
Rem.Energy (%)	[0, 1]	Concave (min.)	(>) ➔ ([0, 1], “min.”, “>”)
1/Rem.Energy	[1, 255]	Additive	(<) ➔ ([1, 255], “+”, “<”)

Moreover, two bit of “T” flags of the RE metric container [10] can be used to represent the node powered mode types, and each DIO message with this information could be disseminated by its sender. The receiver nodes can select an optional routing path through an appropriate routing strategy to avoid using the battery-powered node. The optional Type-Length-Value (TLV) can be used to record the count of these three types of nodes. For example, the count of battery-power node is able to be described as an accessory routing metric organized in additive aggregation rule and “<” as its order relation. Namely, a routing path with fewer battery-powered nodes is preferred. Meanwhile, the counts of main-powered nodes or energy-scavenger-powered nodes can be also considered and carried in the TLVs. However, the domain of this metric is not compatible with ETX and (1/RE), so it only can be used in lexicographic manner based on the theorems of [37].

The other resources of A-LLNs device, such as affordable workload, hardware robustness and availability information, can also be considered as supplements of the energy-aware metric. Three new routing metrics can be piggybacked in the link color object container of a DIO message in RPAL:

− The definition of affordable workload is inspired by the battery index [45] that represents how prone a node is to consume energy. In most cases, this metric will be highly dependent on the node localization, but its computation can be generalized by the following four operating states of a radio transceiver: transmission (TX), reception (RX), idle and sleep. In other words, this metric is a hierarchical Radio Duty Cycle (RDC) since almost all the discrepant energy consumption is associated with the radio operations;
− The hardware robustness is presented as a hardware restart count since the system starts working (*i.e.*, the record provided by NanoRisc on Ext_Milive board [71]);
− The availability information is another resource which represents particular RPAL DODAG paths associated with the application data of interest (*i.e.*, sensing environmental data or event detection) requested by the precision agriculture monitoring application. Namely, this metric can hold the features in a routing path, particularly the role that can mark important retrievable resource information.

In addition, as most of A-LLNs nodes have similar environmental sensing mission workloads, the RDC value is the most influential factor of the affordable workload metric and it will only be taken into account when the DODAG meets routing oscillations, and also when the weight value change between two ETX and RE composite routing metrics is bigger than a threshold. In A-LLNs, restarting is a common operation when the system meets a fault or exception at the software or hardware level. A specific component like NanoRisc [71] is able to provide counter information with a defined sampling frequency.

**Figure 3:**
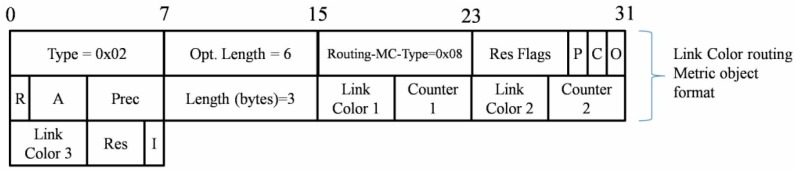
Link color metric and its piggyback format.

Link color routing metric is an efficient metric to disseminate the link quality information. In particular, we note that research about this metric was quite scarce before the drafting of this article. As the ETX metric has been adopted as a link quality indicator, the link color metric container can give three new defined routing metrics a piggyback. In Figure 3, the format of this container using a cumulative color counter is depicted, and the utilization of each Link Color flag field is depicted in Table 4.

**Table 4:** Utilization and explanation of the three link color elements.

Link Color	Carried Data	Utilization
Link color 1 + Counter 1	Affordable workload	If the targeted node is battery powered, the 4-bit of *link color 1* flags will be used to represent the RDC level of this node. Setting low-order bit means RF workload is low and setting high bit for high RDC level. *Counter 1* is used for counting the number of nodes that are too busy in this path.
Link color 2 + Counter 2	Hardware robustness	The 4-bit of *link color 2* flags are used to present the four robustness level of the targeted node. If the restart count is low, the low-order bit will be set. If the node fails frequently in a period, the high bit will be set. *Counter 2* records the number of nodes which are fragile in this optional path.
Link color 3 + I flag	Availability information resource	The 4-bit of *link color 3* flags are used to present four availability information resource (sensing capability) levels. Namely, if this level is high, this targeted node has more monitored info to forward and even need to respond to the queries from sink node. *I flag* is set when that links with the specified color must be included. When cleared, it means this color must be excluded.

Two counters are designated to record the number of the nodes which are too busy and too vulnerable. Additionally, the “I” flag of link color 3 could also determine whether to exclude the links with a specified color. Namely, the nodes, which have important monitoring missions or should respond to the queries in a short time, are able to avoid being as routers or joining a long inefficient routing path. These three link colors present the conditions of A-LLN node in an optional link path so that some dangerous path can be pruned. Essentially, Link Color is used as a heterogeneous constraint rather than a scalar metric like ETX and RE.

To sum up, the purpose of the composite routing metric is to introduce context awareness in the RPAL DODAG construction with 17 bytes more overhead in DIO messages. The dynamic context requires routing metrics and constraints to be described and disseminated for making optimal routing decisions, so RPAL is expected to deal with the real world environment more wisely using resource-constrained hardware.

#### 4.2.3. Context-Aware Objective Function Design

The RPAL model can capture the targeted attributes of node state, link quality, and indications of connectivity intermittence in an A-LLNs scenario. Essentially, SCAOF gives a fundamental rule that defines how to use context-aware information to calculate rank and preferred parent selection. Thus, SCAOF can manage the initial construction and occasional updating of a RPAL DODAG tree. It is particularly designed by composite routing metric/constraints and this is a highly scalable and flexible approach. Therefore, SCAOF is able to employ standardized MrhOF in a more context-aware manner. A routing topology is expected to assign a more important role to the nodes that can positively reply to the requests from an A-LLN application, through using link-quality-aware and energy-aware metrics to avoid the dangerous nodes to forward sensory data, and improving the network energy balance.

SCAOF adopts accumulation, estimation, and prediction techniques over the context from A-LLN sensor nodes to calculate the Instantaneous Suitability *IS_i_* of a node *i* and decides whether to select this node as a parent based on its survivability in the current RPAL DODAG topology. The survivability can be represented by residual energy, link quality, connectivity (duty-cycle), robustness and available information resource of this optional parent. Furthermore, to fulfill the support of the functions and requirements of wireless multi-media sensor network [37,66] (WMSN), we propose to use a lexical approach to contain two lexical elements, and the second part is an additive metric composition function, to express the combination of above metrics/constraints:

(2) $$\left(\text{Link Color Object},\;<\left(\alpha_{1}*\text{ETX}\right)+\left(\alpha_{2}*\text{RE}\right)>\right)$$

This expression currently only supports the IEEE 802.15.4 low-power communication medium. A-LLN sensor nodes will select their parents with appropriate Link Color Object (LCO), and then, the primary routing metrics ETX and Remaining Energy (RE) will work in the additive manner, for ensuring the QoS and network lifetime requirements. A-LLN sensor nodes will calculate their attributes locally and exchange them via DIOs. Then, each node computes the suitability weights of its neighbors respectively, and decides which one can be the preferred parent.

Two LCO rules are defined: to be used as a constraint or as a recorded metric. LCO will bring more flexibility to this metric/constraint composition. We use it to mark whether an endpoint can be a parent or a path is appropriate to forward data packets. As the existence of the first lexical element, the receivers who hear the DIO carrying a LCO will firstly inspect its content. A viable solution of this inspection process is to determine if the suitability values are within the tolerated difference rather than beyond the defined thresholds between two optional nodes or paths. Thus, the first part of the *IS_i_* result can be obtained from the metric/constraint indicated by the counters and “I” flag in LCO:

(3) $${\text{p}}_{\text{pref}}\left(n\right)=p\in P_{n}|\text{min}\left[rank\left(n,p\right)\right]$$

(4) $$rank\left(n,p\right)=rank\left(p,p_{pref}\left(p\right)\right)+\alpha_{1}ETX\left(n,p\right)+\alpha_{2}RE\left(n\right)$$

For the second lexical metric, its suitability equation is similar to the aggregation rule of the additive routing metrics, and its results correspond to their weight parameters, which are also the main factors in the rank calculation of SCAOF. Recalling the aforementioned applicability analysis, precision agriculture applications focus particularly on prolonging network lifetime more than maintaining the link reliability. Thus, the parameters $\alpha_{1}$ and $\alpha_{2}$ here are (0.4, 0.6) or (0.3, 0.7) which will be more fit for PA applications and represent that the A-LLN sensor nodes tend to choose their parents with more residual energy. A generalized method of rank calculation is illustrated in Equations (3) and (4).

It should be noted that different applications have various QoS and constraint requirements. This proposed structure can be used in most of the generalized cases. Furthermore, since the downward routing is built upon the existing DODAG topology, the above proposal is focused on DIO message format and upward routing decisions. On the one hand, environmental data collection by an A-LLN requires a stable converged topology. On the other hand, if the topology for data collection in A-LLNs is well optimized, a bi-directional communication can be easily achieved with appropriate radio devices and symmetric link quality.

## 5. Validation of RPAL SCAOF in Simulations

In this section, the RPAL model and SCAOF will be evaluated in both simulation and hardware testbed scenarios.

### 5.1. Adaptation and Improvement of Simulator, Protocol Stack and Application

The COOJA cross-level simulator platform is adopted in order to evaluate the above proposals. Thanks to the TI MSP430x MCU emulator models, it could help install a full version of the uIPv6 protocol stack with RPAL model and perform evaluation experiments [72]. Therefore, this simulation can provide cross-level debugging for IWoTCore boards [71], and parts of the MiLive WMSN platform [73].

Moreover, the IEEE 802.15.4 PHY layer is the foundation of the IoT network stack recommended by the ROLL WG [74]. It will be the only available choice before the standards of low-power Wi-Fi, Bluetooth and Power Line Communication (PLC) are ready. ROLL WG had a clear statement about IEEE 802.15.4 MAC layer, especially its cluster topology and beacon-enabled mode, which are not well suited for LLNs because these two mechanisms are extremely power consuming due to network management and resynchronization procedures. From the perspective of low-power networks, radio transceivers must be switched off as long as possible to save energy, so the MAC layer solution in Contiki has been adopted and validated by RIME stack [75] and IoT compatible uIPv6 stack.

To prove the RPAL routing protocol can be adapted to agricultural applications, a compatible application protocol is required to equip A-LLNs with WoT technologies. More importantly, the affiliation of CoAP, RPL, IPv6 and 6LoWPAN can be evaluated in the constrained environment of A-LLNs. CoAP is a RESTful application layer protocol which is designed for low-power embedded networks and considered energy, memory and processing constraints of wireless embedded devices [76]. As precision agriculture applications have multiple services, this evaluation work will also present the performance results when a specific application is considered as a WoT use case [77] from the perspective of network protocols rather than building an agricultural web model. We choose the CoAP implementation in Contiki OS which can be applied rapidly [78].

**Figure 4:**
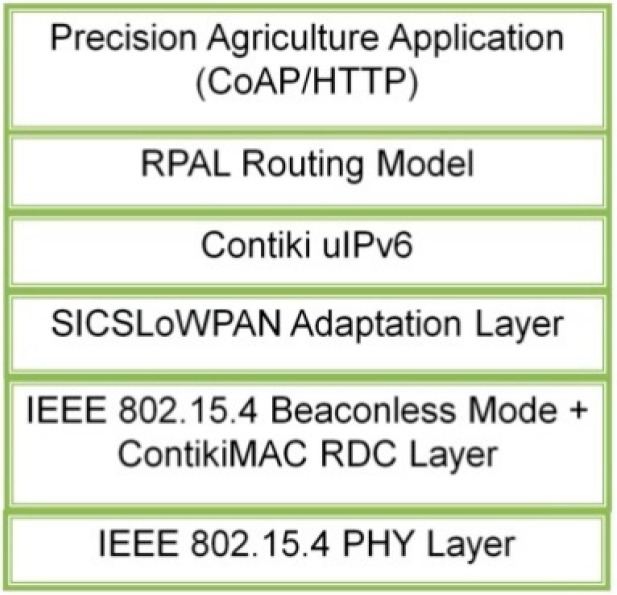
Protocol stack for evaluating the RPAL routing model.

To sum up, the accessions of 6LoWPAN and CoAP enable better evaluation of the RPAL model since an incomplete netstack without low level layers (*i.e.*, IEEE 802.15.4 PHY/MAC and RDC protocol) or high level layers (*i.e.*, 6LoWPAN, IPv6 and CoAP) will not be available to support the full functions of RPAL. Thus, a complete netstack should be adopted (Figure 4). Moreover, due to this flexible netstack, the RPAL model can cooperate well with other emerging protocols and models which are the driving forces pushing traditional WSN technologies into the era of IoT in the agricultural domain.

### 5.2. Simulation Setup and Designated Scenarios

In this section, the configuration of the COOJA simulator, MSPsim emulator and wireless channel model will be presented. Then, the validation of SCAOF in RPAL model will be carried out for different network topologies and different scenarios. Furthermore, the SCAOF algorithm is tested under enhanced simulation scenario scripts which enable runtime adjustments of radio medium, node state and behavior. To the best of our knowledge, this is the first work to evaluate the RPL-based protocol with dynamic simulation and runtime fault injection.

#### 5.2.1. Topology

The majority of existing WSN deployments in field tests adopt a star topology (e.g., a coordinator node as a central node in ZigBee PRO network). A single A-LLN is a centralized architecture, but it is also possible to divided it into a number of smaller structures or extend it with other LLNs through the inter-domain manner of RPL. A-LLNs are expected to be a part of the smart agriculture infrastructure in futuristic scenarios, and there should be a central unit (*i.e.*, an A-LLN border router or edge router) that plays the role of a network coordinator among the A-LLN nodes and the external “things”. Thus, our study is focused on the routing optimization in a single A-LLN so the simulation will be also dedicated to a centralized architecture.

The assumptions of a grid-like network topology as well as a tree-like network topology are common simplifications. In some cases, these two types of topologies could be equal for DODAGs organization of RPL framework. The difference in them is that the main consequence of the former is a near-uniform distribution of the number of node degree (*i.e.*, neighbors’ count). Distance Loss Unit Disk Graph Medium (UDGM) in Cooja is often used to build simplified scenarios configuring radio coverage and distance loss as fixed parameters [37,38,79,80], and here UDGM is adopted to evaluate RPL and RPAL.

Since realistic crop fields are mostly organized in the adjacent regular parcels, the shapes of them could be rectangle or quadrangle (see Figure 1), so they are similar to grid topology. Considering the network scalability as well as the low density of the A-LLN, the number of sensor nodes is defined as 20 and 30. The sensor node locations are decided to be the junctions of the edges of adjacent grids or the centers of the grids according to the above assumptions. Namely, they are uniformly distributed around but the sink node is placed at the boundary of this topology.

#### 5.2.2. Traffic Pattern

Essentially, A-LLN nodes adopt UDP as the transport layer protocol and their application process includes best-effort transmission and acknowledges. Therefore, the traffic in an A-LLN is distributed evenly between P2MP (*i.e.*, DIO and DIS multicast and IPv6 Neighbor Discovery mechanisms) and MP2P (*i.e.*, application packets and DAO transmission). It is important to note that CoAP will carry out the queries of available information resource in the unicast manner. In particular, the traffic pattern of each A-LLN node depends on the logical roles in Table 5.

**Table 5:** Traffic patterns of the nodes with different logical roles in an A-LLN.

Node Type	Supports of Traffic Pattern
**A-LLN Edge router/border router**	Sending a resource query request as 5 CoAP packets burst to an actuator in 60~90 s interval; ACK of received frames;
**Common monitoring A-LLN sensor nodes**	Periodic reporting in 25~30 s interval
**Local controller/Actuator**	Period reporting in 10~15 s interval; sending ACK; sending resource query reply packet to edge router
**Malicious sensor nodes**	Periodic reporting in 25~30 s interval

Note that the reporting periods are not similar to the realistic reporting frequency that could be only two to ten times per day, but the above node role design of general traffic patterns is very similar to a real world system. Furthermore, all the A-LLN sensor nodes are able to turn their node types to malicious ones.

#### 5.2.3. Simulation Parameters

All the nodes in this set of experiments run Contiki OS on an emulated platform built by MSP430x MCU with enough RAM memory and IEEE 802.15.4 compliant radio CC2520 module. The MSPSim hardware emulator is able to introduce hardware constraints into this simulation. Powertrace model is adopted to monitor the energy consumption of each node. To observe the comparison results, the configurations of different layers of Contiki uIPv6 protocol stack are set to the default parameter states.

See Table 6 for a detailed description of the network and the simulation configurations. The simulation output is recorded in the raw logging files for statistical analysis. For each simulated scenario of different network size, five simulations are applied repeatedly then five random topologies per network scenario and the final results are computed as average values.

**Table 6:** Network and simulation configuration for validation of SCAOF.

Network	
Deployment area	25 m × 20 m
Deployment type	Random positioned
Number of nodes	1 sink with 20 or 30 sensor nodes
Radio coverage	100 square meters
Distance loss	90% RX Ratio
Nodes initial energy	0.25 mAh = 2700 mj; millionth of 2500 mAh estimated by PowerTrace Model with assumed stable 3 V voltage
Network layer protocols	uIPv6
Routing protocol	RPL routing framework: Trickle timer: k = 10; IntervalMin = 12, IntervalMax = 8; Routing Metrics: ETX, RE, link color
Transport layer	UDP
Data link layer	CSMA/CA + ContikiMAC + 6LoWPAN
**Application**	
Data length	20 bytes per packet
Task type	Time drive
Reporting intervals (s)	15
**Simulation**	
Time	40 min
Iteration	5

### 5.3. Validation of Energy-Aware Routing Metrics and SCAOF Performance

The RPL and RPAL models are validated in terms of network lifetime, packet delays, packet loss rate, Round-Trip Time (RTT) for CoAP resource request/reply, routing table size, overheads and path hop distance. For testing these performance metrics, we built two simulation scenarios.

#### 5.3.1. Network Simulation Scenarios: 20 and 30 LLN Nodes

In this scenario, 20 and 30 battery-powered A-LLN sensor nodes are respectively simulated in a grid topology. The comparison is drawn between the standard RPL model and the RPAL model. The performance metrics of network lifetime are recorded when the outputs of the Powertrace model arrive at the predefined energy storage threshold. Whether a node is active depends on the existence of a connection to the sink node.

Average data collection packet delay is measured as an average value representing the time cost of the sender node to send messages to the sink node successfully. The relevant distance to the sink node for each sensor node is presented as rank in the DODAG. Because rank is not a stable value in the procedures of DODAG maintenance, it is used as a snapshot value when the nodes run out of energy.

Table 7 and Table 8 present the results from the simulation testing and quantify the performance difference between the SCAOF with energy-aware routing metrics and standard RPL MrhOF with ETX primary metrics. In Table 7, the network lifetime is considered in the two cases mentioned previously. The difference in lifetime is not large, because a small amount of energy was assigned to each node. This leads to an unexpected result that some nodes of SCAOF cannot afford the energy consumption for a parent reselection when the time is near the end of the simulation. Nevertheless, lifetime performance of RPAL’s SCAOF is better than RPL’s MrhOF in most comparison items for testing, except in the case where the percentage of alive or active nodes equals 0%.

**Table 7:** Network lifetime of 20 and 30 LLN nodes scenarios.

**Performance Influenced by Using RPAL SCAOF**	**Performance Metrics (+: Increase, −: Decrease)**						
	**Lifetime**						
	First dead node (min)	% of living nodes = 50% (min)	% of active nodes = 50% (min)	% of living nodes = 30% (min)	% of active nodes = 30% (min)	% of living nodes = 0% (min)	% of active nodes = 0% (min)
**20 nodes**	+3.4	+1.25	+7.63	+2.25	+12.17	−4.75	−3.53
**30 nodes**	+3.03	−6.75	+1.58	−7.51	+1.81	−6	+4.28

For other network performance metrics (*cf.* Table 8), it is evident that OF using ETX primary metrics will select the shortest path, while a routing metrics combination solution is able to choose a longer path (*cf.* “Path hop distance” in Table 8) to the sink node for avoiding the energy depleting node. This may lead to higher cost in packet delay, overhead on consumed memory and control messages. As SCAOF is dedicated to keeping more nodes in an active state, it can achieve a lower average packet loss rate because busy routers have a longer lifetime in these tests.

**Table 8:** General performance metrics of 20 and 30 LLN nodes scenarios.

Performance Influenced by Using RPAL SCAOF	Average Data Collection Packet Delay (ms)	Average Packet Loss Rate (%)	Average Number of Route Entries	Control Plane Overhead (bytes)	Average Path Hop Distances	Average CoAP RTT (ms)
**20 nodes**	+34	−3.62	+0.87	≈ +2541	+0.61	−124.37
**30 nodes**	+38	−9.18	+0.88	≈ +3724	+1.71	−110.7

Apparently, as the network size and density are increasing, more redundant RPL routers will exist in the DODAG. This leads to more serious hotspot issues when the standard RPL is adopted. In fact, more nodes cannot maintain the links with the sink node although more nodes are alive, but essentially they cannot participate in the business of this network.

Table 8 reveals the network performance metrics of this simulation. The trade-off between the network lifetime and certain network performance is still like the last experiment. It should be stressed that 20% of the simulated LLN nodes have the CoAP application installed so they are able to reply with resource information organized by the CoAP protocol, and sink nodes will periodically transmit resource requests to these six nodes. The average CoAP Round-Trip Time (RTT) is the result we got from this simulation, and it proved that our Link Color metric is able to improve the communication of the nodes running CoAP applications by impacting the DODAG topology rank.

The costs due to SCAOF become bigger as the number of nodes is increasing, but the results would be better if the nodes are assigned as normal energy level since the small volume of energy introduces very sensitive calculation of routing metrics that might lead to frequent parent reselections. Although the network churns only happen in local areas, RPAL model will certainly trigger local repair mechanisms more times than the original RPL protocol. Furthermore, SCAOF still can achieve lower packet loss rate as its composite routing metric approach could balance the traffic (*i.e.*, the routing metric brings RDC information) as well as avoid energy depletion (*i.e.*, RE routing metric). Furthermore, a certain level of delay for the common periodical application packet transmission is not a serious issue compared with the network lifetime performance as A-LLNs are delay-tolerant networks.

#### 5.3.2. Network Scenario: 30 LLN Nodes with Runtime Reconfiguration of the Node State

To evaluate the performance benefits brought by our proposed SCAOF and composite RPAL routing metrics, we have run a specific scenario with different levels of penetration of misbehaving nodes randomly distributed in the grid that is like previous experiments. These misbehaving nodes perform periodic resets of their watchdog that is implemented by controlling the emulator to trigger this behavior. After one system restart time stamp, these nodes will try to rejoin the DODAG, meanwhile they also can be seen as the sources of a “grey hole attack” since they will randomly drop the received traffic during the time when the emulated devices start the bootstrap program until they are ready to be a member of their joint DODAG.

To simplify analysis of the results of this experiment, we provide enough energy to all the nodes for running a 40 min simulation. A JavaScript-based simulation script is used to give the runtime reconfiguration of the node state and generate random restart intervals for those misbehaving nodes. The obtained results regarding the packet loss, latency, number of failed co-operations, and packet transmission cost, are listed in Table 9. From the results, we can observe that the original RPL cannot solve the problems caused by the misbehaving nodes. Its packet loss increases very quickly if the number of misbehaving nodes is increased, and even though the ETX routing metric is able to alleviate the unreliable links its cumulative feature will still cause long-duration delays for carrying out these measurements. Thus, since RPL’s MrhOF does not have any mechanisms to promptly detect the misbehaving nodes, more failed packet forwarding will happen, and the cost for the successful delivery of one data packet to the sink node is higher than in RPAL. In the composite routing metrics solution, the Link Color metric is able to reflect the robustness of the RPL router device and SCAOF sets this metric as a precondition when a node selects its parent. Misbehaving nodes can actively broadcast their node state to their neighbors. This evaluation can prove that this is a better method than waiting for the other nodes in the normal state to monitor these dangerous routers. In other words, SCAOF can prevent the A-LLN nodes from connecting the unstable nodes but it needs to select a longer path, and this is the reason why we obtained higher latency results in the performance comparison.

**Table 9:** Simulation results of 30 LLN nodes with node state runtime reconfiguration.

Penetration of Misbehaving Nodes (%)	Performance Influenced by Using RPAL SCAOF (+: Increase, −: Decrease)			
	Average Packet Loss rate (%)	Average latency (ms) of successful transmission	Number of failed co-operations for packet forwarding	Packet transmission cost
10%	−11.43	+9.53	≈ −746	−1.09
20%	−21.52	+20.53	≈ −1156	−2.13
30%	−33.56	+21.08	≈ −2200	−2.24

It should be noticed that considering the robustness of device as a context feature as well as routing metric, a specific multi-core hardware platform like IWoTCore board [71] can provide accurate statistic results of the node state (*i.e.*, times of restarting, power management, *etc.*), so that SCAOF could obtain the required context-aware data from the hardware level, which is able to greatly increase the efficiency of this OF algorithm.

## 6. Evaluation of RPL and RPAL in a Real World Environment

To validate the results from the performed simulations, we performed a set of tests on a real-life testbed (IWoTCore plus Ext_MiLive extend board [71]). For these tests, the maximal number of retransmissions was increased to three since some links in the field test were less reliable and the link qualities are extremely dynamic due to obstacles (e.g., trees, buildings or barriers). For the wireless multi-hop communication in these tests, radio transmitter modules were tuned using channel 19 and a transmit power of 3 dBm (the highest setting level in the RF230bb’s driver).

### 6.1. Testbed Setup

**Figure 5:**
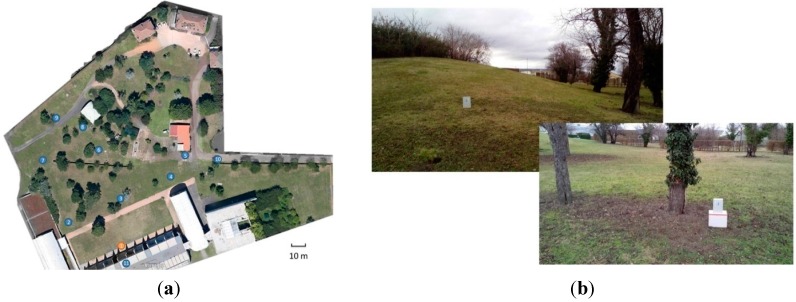
(**a**) A plan of testbed deployment; (**b**) Testbed setup: photos of No. 2 and No. 3 deployed IWoTCore node.

The localization of the deployed nodes is shown in Figure 5a. It is a prototype of an environmental data collection system based on the Contiki OS and uIPv6 protocol stack, which is located in the garden of the IRSTEA research center (Aubière, France). 

**Figure 6:**
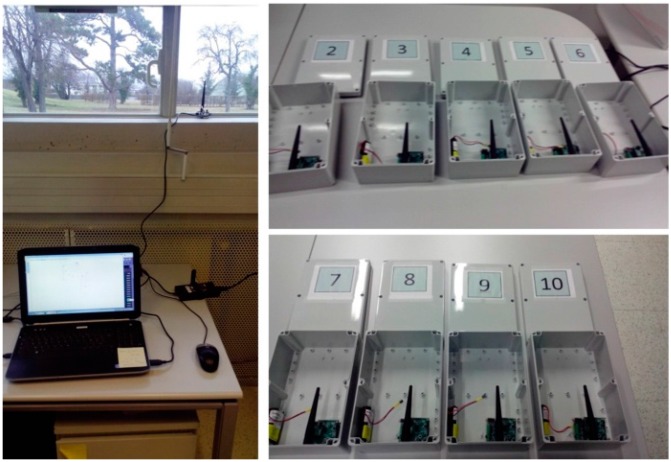
Testbed setup: sink node and nine IWoTCore Ext_MiLive nodes for outdoor environment experiments.

Currently, this testbed consists of 11 IWoTCore core nodes with Ext_MiLive extend boards, which one of them is directly connected to a laptop (see Figure 6) through a USB port as the sink node, and an adapted version of a collect-view application is running on the laptop for observing different types of measured data. The main objective of this testbed is to monitor the viable states of network, hardware, power supply, and scalar sensing data (air temperature and light intensity). It is to be noticed that one IWoTCore node (tagged by 11 in Figure 5a) is located in an office as a contrast experimental object.

To run this testbed system in the real world environment, the boards and their rechargeable power source (Li-Ion Battery Pack from ENIX ENERGIES, Saint Egrève, France) are well encased in hermetical plastic containers (see Figure 6). As shown in Figure 5b, the testbed devices are located where the deployment situation has a certain geographical gradient (little to medium incline) as well as numerous obstacles, like walls, buildings, shrubberies, trees, and facilities in the garden. To increase the reliability of transmission, the antenna height is temporarily raised to 45 cm for the testbed devices deployed in the low gradient level area as depicted in Figure 5b.

During the setup of this testbed, from the empirical results of our tests, if the antenna’s height is lower than that of the obstacles, link quality is very dynamic because the transmission would be mainly based on Non Line of Sight (NLOS) signals, and the link quality would be seriously affected by phenomena like reflection, refraction, diffraction or scattering. The radio chips have to be configured with the same settings like transmission power and identical hardware behaviors, so that most of the radio links are symmetrical in both directions. Even so, the radio channel is intrinsically unstable due to the impacts of various environmental factors and interferences. The easiest observation is that some links can occasionally disappear or reappear, and this phenomenon would imply certain dynamics in the network. Thus, keeping all the testbed devices in a homogeneous situation is significant for the later comparative tests. To deploy the prototype system in the real world environment and organize three comparative experiments, the following simplified considerations and corresponding explanations are defined:

− Testbed node 1 is the sink node connected to a laptop and used as a data collector and remote controlling message emitter.
− Deployed nodes are located at the same positions, at the same relative angles and distances.
− This prototype system could provide three categories of required measurements (see Table 10).
− The other 10 nodes would have different power supplies, but with the similar experimental environments, such as weather condition, duration of test, data collection frequency, and state of charge for all batteries, in three comparative experiments as listed in Table 11 and Table 12.

**Table 10:** Three main types of required measurements for statistical analysis.

Required Data Type	Measurements Gathered from the Testbed
Sensor data	Temperature; output of battery voltage; restart counter; and light intensity (**Depends on transparency of the utilized waterproof plastic box**)
Network states	Size of neighbor list and routing table; topology; controlling message interval; ETX value; Rank value; packet delivery ratio (PDR); number of hops; number of churns
Power supply states	Average power consumption; average radio duty cycle; battery indicator from online energy estimation model.

**Table 11:** Similar settings in three comparative experiments.

Experiment Settings	Details and Parameters
Collecting frequency	60 s–120 s
Duration of test	6 h (expressed as 1:00 to 7:00)
Initial energy of power supply	594,000 mJ (10% of nominal capacities in battery’s fully recharged state) When the battery is depleted, the radio chip is off.
Heavy task for fast energy consuming (reduce 70% battery)	The testbed node 3 and 6 pretend a 70% decrease of their remaining energy by manual remote control application at [3:55, 4:00].
Testbed node with Misbehavior of restarting	Testbed node 4 has communication problem with its NANO module within a frequency of 600 s–1200 s during the periods of its lifetime.

**Table 12:** Different settings in three comparative experiments.

Sequence N. of Comparative Test	RPL Model	Routing Metrics	Testbeds with Energy Harvesting Module (Solar Panel)
1st experiment	Standard RPL model	ETX	No
2nd experiment	RPAL model	ETX; Context-aware metric	No
3rd experiment	RPAL model	ETX; Context-aware metric	Yes (testbed node 3 and 6 recover their batteries from 4:00 to 5:00)

− To explain the consequence of introducing misbehaving nodes, the concept and utilization of NANO module needs to be clarified. It is a specific energy efficient SCM and its designed program is used to guarantee the robustness of the targeted system. The mechanism is to force the software running on the AVR MCU to keep periodical communication (a loop of state reading) with the NANO module. If this rule is broken, the whole system will be reset and the interior counter of NANO will be increased to record this restart behavior of the system.
− As three comparative experiments should be conducted in the same scenario, the weather conditions and system problems are essentially unpredictable, and the unbalance of energy consumption requires long-time accumulation, thus, a remote controlling application is implemented for sending commands (see Table 13) to achieve the expected settings of different tests. To ensure the command packets are well received, a repetition mechanism is performed until the receiver replies with an ACK message.

**Table 13:** Remote control and fault injection functions.

Functions	Descriptions
**LED control**	ON and OFF switching the single LED on IWoTCore board.
**Message collection**	Prepare and send a collect-view application packet immediately.
**RPL global repair**	Trigger global repair in the current DODAG.
**Collecting frequency control**	Change the frequency of sending collect-view application packet to 10 s, 15 s, 30 s, 60 s, 120 s.
**Remained energy control**	Modify the volume of battery +10% and −5%. The results can be observed in the battery indicator plot.
**NANO control**	Postpone the event timer of the NANO communication process.
**TX power control**	Modify the transmission power of the radio chip to a designated value.
**Power supply mode control**	Configure the targeted testbed using the below power supply modes: Mode 0: battery powered, residual energy is based on online energy estimation model Mode 1: energy harvester module (solar panel) is able to produce enough power to activate the testbed and cannot recharge the battery Mode 2: energy harvester module (solar panel) is able to produce enough power for both testbed routines and battery recharging.

### 6.2. Evaluation Results

The objective of the three comparative experiments is to reveal the characteristics of an IoT prototype system under different routing strategies. The comparison results can be obtained, especially the influence of the standard RPL protocol or RPAL protocol with SCAOF and composite routing metrics. Firstly, the effects of introducing fast energy consuming node (3 and 6), and misbehavior node 4 will be evaluated. Moreover, the performance results of the 1st experiment will be compared with the 2nd one.

#### 6.2.1. Number of Hops 

**Figure 7:**
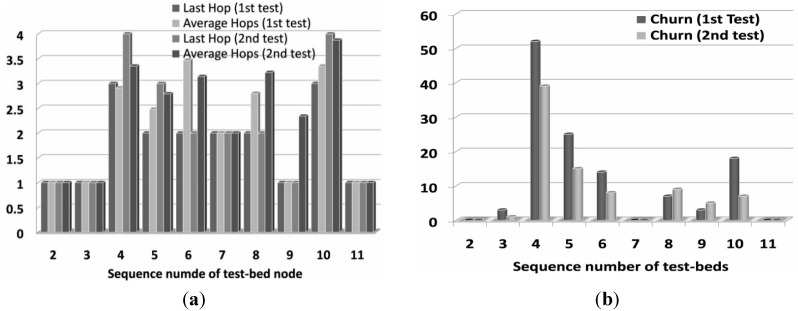
(**a**) Hop count evaluation results; (**b**) Number of network churns for the testbeds in the 1st and 2nd experiments.

Figure 7a shows the results of average hops and last hop in the 1st and 2nd experiment. As nodes 3, 4 and 6 are important routers in this network, and they will encounter serious problems during the experiment, such as battery depletion and system unreliability. The augmentation of hop number is an easily observed result to evaluate whether the tested routing strategy can comply with the corresponding answers. The RPL standard protocol only uses ETX as routing metric for calculating the minimum rank with hysteresis OF. Thus, according to the measurement data, the RPL model in the 1st test has less hop counts since it didn’t change routing path before node 3 and 6 consumed all their battery energy. Nevertheless, the RPAL model has the ability to move the exceptional nodes to a lower rank position which may lead to larger rank value and more hops count in the 2nd experiment.

For the case depicted in Figure 7a, node 4 has more hop counts in the 2nd test than in the 1st test, because its NANO module counter value is advertised by its emitting DIO messages as a routing constraint and its neighbors will not choose it as preferred parent. Meanwhile, this also leads to increased hops counts for nodes 5 and 10.

#### 6.2.2. Network Churns

In Figure 7b, the statistical oscillation count of the targeted network is depicted. Since the captured DODAG structures during the test cannot all be listed here, the number of network churns is adopted to present the stability of the observed DODAG topology.

The results prove that the RPAL model is able to reduce network oscillation compared with the original RPL model. The main reason is that standard RPL protocol adopts ETX to find high throughput paths. Since ETX is always searching for the best instantaneous link quality and the packet delivery ratio (PDR) estimated by sending probes is a stochastic variable in a real world environment, instability may be unnecessarily induced in our static network, even hysteresis has been used to reduce churn in response to small ETX metric changes. Due to the existence of weight parameters in our RPAL model, the effect of hysteresis is magnified and this decreases the count of the switching parent. In addition, node 4 is used to be a key router in the 1st field test, and its unreliable characteristics will lead to more churns for its potential children (nodes 5, 6 and 10). Last but not least, it should be noticed that the RPAL model may also cause network churns due to its selection of a new routing path to avoid the battery depleting nodes. However, in our case, this impact is very limited and the evaluation results prove that the mechanisms in SCAOF will not produce serious oscillations in a wide range of existing DODAG.

#### 6.2.3. Packet Lost Ratio

**Figure 8:**
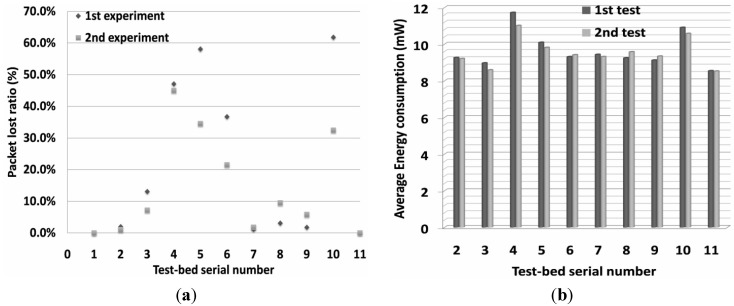
(**a**) Packets lost ratio in the field tests of 11 IWoTCore nodes; (**b**) Average energy usage of the testbeds in the 1st and 2nd experiments.

In Figure 8a, for nodes with an average hop count from the sink node lower than two, there was almost no packet loss detected. This is the effect of configuring with four retransmissions and the existing line of sight propagation. Notice that when the hop count equals to three, the packet loss increases significantly. On the one hand, low packet delivery is caused by the bad link quality of intermediate nodes. For example, monitoring packets from node 5 and 10 have to transverse the optional parents 4, 3 or 6, but node 4 keeps rejoining the DODAG network and this also leads to frequent disconnections when it serves as parent. This causes higher packet loss for nodes 5 and 10 in the 1st experiment than in the 2nd one. Besides, the statistical results also count the part of the packet loss if node 3 and 6 are dead before the end of these two tests. As the SCAOF algorithm has the ability to adapt the network topology to avoid nodes 3 and 6 as busy routers, the results indicate that these two nodes lose fewer packets in the 2nd test. Notice that nodes 8 and 9 have more network churns in the 2nd test and they have more lost packets, since real world network re-convergence normally requires longer time to achieve. On the other hand, the interference due to other wireless equipment (*i.e.*, office Wi-Fi or a weather station in the case of the garden at IRSTEA) and the signal attenuation by passing through obstacles near the area where the testbeds are deployed will cause worse packet loss.

#### 6.2.4. Energy Usage

**Figure 9:**
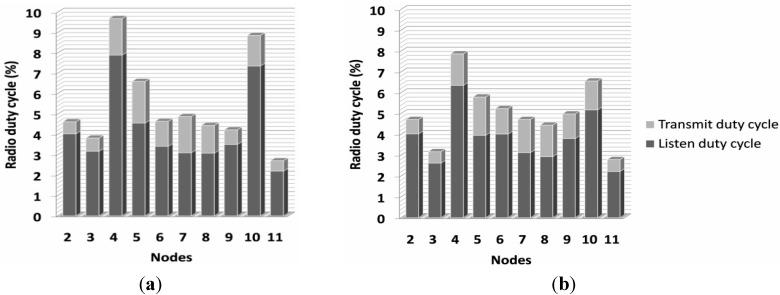
(**a**) RDC results of the testbeds using standard RPL model in the 1st test; (**b**) RDC results of the testbeds using RPAL model in the 2nd test.

In Figure 8b, the effects of two routing models on the energy consumption performance is illustrated. In comparison with the simulation results in Section 5.2, we have to admit that the behavior is different from the expectations. This outcome can be explained by the following reasons: the nodes in the simulation tests have the same link conditions but the field test nodes do not have the same link conditions. In other words, the repair mechanism for detecting routing loops is initiated more often in the real world tests, and this could be responsible for an increase in the power consumption. Furthermore, the current prototype system cannot provide low MCU power mode due to the lack of an appropriate solution using Contiki process management to deal with the correlation between the AVR MCU and the NANO module. Thus, keeping MCU in normal working mode is a safe and temporary method to evade these potential issues. Besides, in our tests, if the radio chip enters sleeping mode or the uIPv6 process is trying to send/receive a packet, and at the same time, the process of reading NANO’s state has a certain level of communication delay, the wake-up interval in this node will become unstable and lose the synchronization with its neighbor nodes, and this leads to the failure of subsequent radio transmissions. Although this is an event with a very small possibility, the tests show that it will happen within the duration of 20 to 30 h. Thus, the RDC mechanism (*i.e.*, fast sleeping and low power listening) in the ContikiMAC model and the transmission of collect-view application packets are designed to have a short intermission when they have conflicts with the NANO process. This could also cause the augmentation of radio duty (depicted in Figure 9).

The RDC results in Figure 9 further prove that the RPAL model can prevent network churns and reduce energy usage due to misbehaving nodes, and improve the topology stability by increasing the effects of hysteresis. For example, node 3 has smaller RDC in the 2nd test after the energy consuming task is carried out, it sinks to lower rank of DODAG and the other nodes will select optional routing path. In other words, standard RPL model will continue a relatively greedier PDR searching algorithm to treat node 3 as the key router. In the case of our designed experiments, the two tested routing strategies will impact the lifetime of these two exceptional nodes (3 and 6). According to the recorded data, nodes 3 and 6 can survive around 31 and 14 min longer respectively in the 2nd experiment than in the 1st one. However, the obtained results cannot ensure the RPAL model is energy efficient since the number of redundant routers and the duration of experiments are still limited, but at least, RPAL outperforms the standard RPL model in the aspect of energy balance, which can lead to longer network lifetime.

As the only difference between the 2nd test and 3rd test is whether nodes 3 and 6 can recharge their batteries from the emulated solar panel, energy power mode becomes the single factor that most impacts the network structure. However, no improved network performance in the 3rd experiment can be observed, except in the packet reception ratio. More network churns emerged compared with the results in the 2nd test. We also notice that the network topology is not stable during the period when the battery-depleting nodes have up-and-down battery volume because they will become optional key routers along with the restoration of their batteries, and this enables them to be selected as preferred parents again.

## 7. Conclusions and Future Work

ICT in agriculture needs the support of conventional WSN technology, and it is hoped that this work will become a driving force to push the use of WSNs in agriculture to the IoT. The A-LLN architecture for adapting the traditional WSN of Precision Agriculture (PA) applications to IPv6 LLN has been elaborated. Considering the ability to resist the highly dynamic environments in the real world, this work has focused on the energy optimization and the robustness of the whole network with a view to overcome the existing challenges. This paper has presented the design, implementation and evaluation of an enhanced RPL-based model RPAL with a scalable context-aware objective function, the SCAOF. The RPAL model integrates energy-aware routing metrics and SCAOF with composite routing metrics, which can introduce context features into the original RPL’s mechanisms. The solutions in this paper can mitigate the hotspot issue, prolong network lifetime and improve the QoS of A-LLN by combining the remaining energy of A-LLN sensor nodes, as well as considering the other factors including RDC, device robustness and information availability, into path weight calculation. The proposals have been compared with the original protocol in a set of simulation experiments, and evaluated in a prototype system deployed in the IRSTEA garden. The obtained results show that the proposed solution could allow for the effective use of resource-constrained devices in the A-LLN scenarios, thereby reducing system costs and improving the availability of IoT technologies for agriculture.
